# Activity patterns of the soprano pipistrelle *Pipistrellus pygmaeus* throughout the year in southern Norway

**DOI:** 10.1186/s40850-021-00065-x

**Published:** 2021-02-09

**Authors:** Karl Frafjord

**Affiliations:** Tromsø University Museum, UiT The Arctic University of Norway, P. O. Box 6050 Langnes, 9037 Tromsø, Norway

**Keywords:** Activity, Foraging, Social calls, Yearly variation, Hibernation

## Abstract

**Background:**

Most temperate bats are regular hibernators in the winter. Knowledge about the length of their active season and how they adjust their nightly activity throughout the season, is critical to conservation. The characteristics of these are likely to vary with climate as well as latitude. This study investigated the flight activity of the soprano pipistrelle *Pipistrellus pygmaeus* in Frafjord, a small valley in the south-western corner of Norway (58° 50′N 6° 18′E) with an oceanic climate.

**Results:**

Activity was recorded with an ultrasound recorder throughout April 2018 to June 2019 at one site, with supplemental recordings in March to June 2020, i.e., covering all months of the year. Recordings at other nearby sites were made in the summers (June–August) of 2016, 2017, 2019 and 2020, as well as some of the last days in December 2019 to the first days of January 2020. Overall, soprano pipistrelles were recorded flying in all months of the year, but very few in December–March. Regular activity was recorded from late April or early May until late October, and some recordings were also made in November. The highest numbers of recordings were made in August and September. Social calls, i.e. male song flights, were recorded from April to November, with the vast majority in August and September. Nearly all recordings were made between sunset and sunrise.

**Conclusions:**

The soprano pipistrelle in this region showed regular activity through 6–7 months of the year. It adjusted its activity to the changing night length throughout the year, closely following sunset and sunrise. It was rarely recorded flying before sunset and almost never after sunrise. Most activity was recorded in the middle of the night, and social calls also followed this trend closely. Harems in late summer and autumn were confirmed in a bat box, which was also used for winter hibernation.

**Supplementary Information:**

The online version contains supplementary material available at 10.1186/s40850-021-00065-x.

## Background

Insectivorous bats have evolved a number of adaptations. Among these are flight, echolocation, nocturnal hunting and hibernation. Several hypotheses have been suggested to explain their nocturnal hunting strategy; avoiding diurnal predators, avoiding competition with insectivorous birds or avoiding high daytime temperatures [1–3]. Among these, predator avoidance appears to be most widely supported [4–6]. Because bats in temperate regions hibernate during the winter, they basically fly in only part of the year. Most of these bats also enter daily torpor as a means to save energy, especially in inclement weather [7]. For females, the costs of pregnancy and lactation are high, and they may prefer to avoid torpor in these periods [7]. Torpor may extend pregnancy, delay birth and reduce milk production.

Pipistrelle bats, *Pipistrellus* spp., are very small and often very numerous. Whereas several studies address the activity and behaviour of the common pipistrelle *P. pipistrellus* [8–15], little is known about other pipistrelle species [16, 17]. The common and soprano pipistrelles *P. pygmaeus* were, for example, only identified and separated in the 1990’s [18]. Earlier studies were of uncertain species, although it is likely that most involved the common pipistrelle.

The soprano pipistrelle is common in South Norway [19] and appears to hunt in brighter light than most other bats in this region. It may emerge before sunset at 62°N, but then it hunted inside the forest and not in the open until the night became dark [20, 21]. This contrasts with the behaviour of the common pipistrelle, which emerge well after sunset [8]. Russo et al. [22] verified this difference between the two species and confirmed that forest habitat was used by early hunting soprano pipistrelles. In other habitats the soprano pipistrelle may emerge later and after sunset [23].

Hollow trees or rock crevices are probably the natural nursery roosts of the soprano pipistrelle in Norway, but when available it prefers warmer, man-made structures such as buildings and bat boxes [24]. Weather influences pipistrelles in a multitude of ways, both in their ecology and behaviour [17, 25]. The common pipistrelle may hunt also during the winter months, particularly in warm and calm weather [26].

Male pipistrelles of all species have characteristic “mating” displays, using particular vocalisations and behaviours to announce their presence and to attract females [27, 28]. These vocalisations are called social calls and are emitted by both common and soprano pipistrelle males during prolonged song-flights. However, Barlow & Jones [29] concluded that these social calls were used in food defence when insects are scarce rather than in social displays [30].

This study describes the nocturnal activity of the soprano pipistrelle throughout the year and through the night. The main aims were to study how this bat adjusts its activity to the changing night length and the timing of the males’ social calls.

## Results

**Fig. 1:**
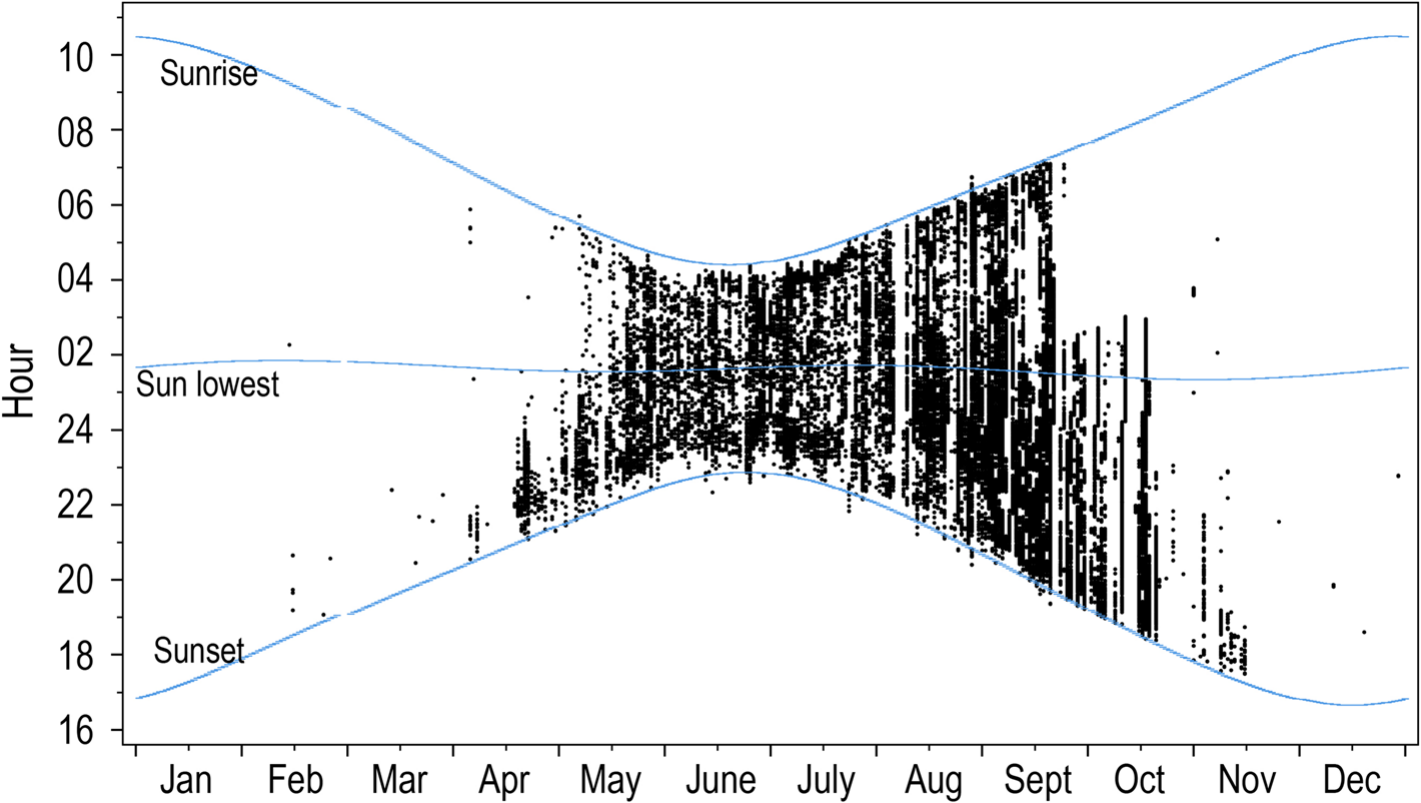
Distribution of soprano pipistrelle recordings during the night across the year in Frafjord, SW Norway. The lower line represents sunset, the upper line represents sunrise and the central line represents the sun at its lowest angle. Each point represents a 1 min interval in which bats were recorded. Note that many points are superimposed. Recordings were not made in the last part of the night from the last days of September to December

The sample sizes were: echolocation recordings of soprano pipistrelles at the main site *n* = 20,732 min, social calls at the main site *n* = 7036 min, and echolocation recordings at other sites *n* = 3194 min. The first soprano pipistrelle in 2018 was recorded in February (Fig. 1). Very few were registered in February, March and December, and relatively few in April and November (Table 1). Numbers increased from April to September, and declined in October and very much so in November.

**Table 1:** Earliest and latest times (local summer time, 24 h clock) soprano pipistrelles were recorded by month

Month	First time	Last time	N min	Lux in 22 h	Lux in 23 h
*Main site*					
Feb	1904	0216	7		
Mar	2027	–	5		
Apr	2033	0553	342		
May	2127	0542	1938		
June	2240	0415	2002	1050	560
July	2234	0458	2052	970	290
Aug	2047	0618	3707	530	70
Sep	1913	0710	7935		
Oct	1751	–	2525		
Nov	1730	–	216		
Dec	1836	–	5		
*Other sites*					
June	2236	0423	629		
July	2150	0512	1207		
Aug	2024	0645	1358		

The times are based on recordings at the main site and other sites in Frafjord, SW Norway. N = sample size in minutes. Maximum lux in the 22 h (i.e. 2200–2259 h) and the 23 h were recorded in Sandnes 2007

To further examine whether the pipistrelle could also be flying in late December to January, some extra recordings were made in the period 22 December 2019 to 4 January 2020 at the bat box. This confirmed flying pipistrelles (Table 2). Additionally, two pipistrelles were observed flying at the farm on 2 January 2020 at 1815 h (8 °C, insects were also seen flying). The ultrasound recordings confirmed occasional hunting (feeding buzzes) in winter. Flights in December 2019 to January 2020 were confirmed by changing numbers of pipistrelles in the bat box, when some of the bats left the box at colder and re-entered at warmer temperatures (Table 2). Hibernating soprano pipistrelles were found in the bat box in all winters from December 2015.

**Table 2:** Changing numbers (N) of soprano pipistrelles in a bat box with ambient temperature (°C)

Date	N	^°^C	Sound
21 Dec	5–7	7.0	
22 Dec			5
23 Dec	7	4.5	2
26 Dec	2	−2.2	
27 Dec	2	2.8	
28 Dec 10 h	3–4	3.9	
28 Dec 23 h	4–5	6.4	
29 Dec	5–6	9.8	4
31 Dec			3
1 Jan	5–6	4.5	1
2 Jan	7	6.8	
4 Jan	7–10	−0.4	

Winter recordings made from 21 December 2019 to 4 January 2020 in Frafjord, SW Norway. The ambient temperature was recorded about 8 m from the box at the time of counting. Sound is the number of minute intervals during which echolocation of flying pipistrelles were recorded by the data-logger

Although pipistrelles could be recorded flying in all months of the year, their main active period was from late April or first days of May until the middle of October (Fig. 1). Outside this period, the recordings were sparse (Table 1). The major increase in spring activity occurred from 5 May 2018 and 19 April 2020. Consequently, the pipistrelles’ main active season lasted 5 to 5.5 months, with a rather abrupt start and end.

At the farm site, pipistrelles were recorded throughout the night and almost every night throughout the main activity season (Fig. 1) with nearly 100% between sunset and sunrise. There was no clear time-lag between pipistrelle activity and sunset or sunrise (Fig. 1, Table 1). Their activity was centred around the time the sun was at its lowest angle, but marginally skewed towards the evening on some nights. This skew could be 10–15 min. The bats adjusted their activity to sunset and sunrise almost perfectly tracking the changing night length throughout the year (Fig. 1). They thus apparently preferred light levels below 1000 lx, possibly mostly below 500 lx (Table 1).

**Fig. 2:**
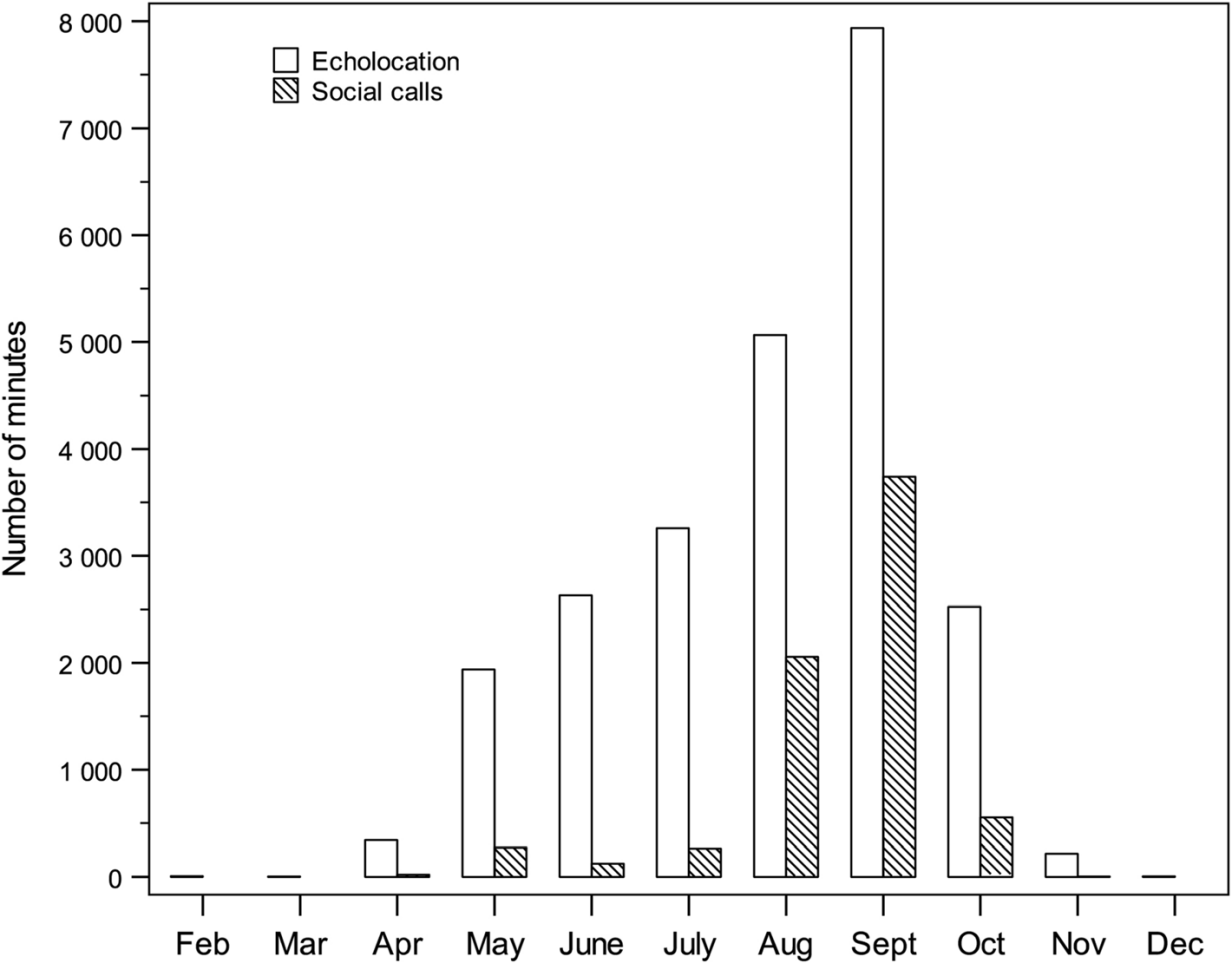
Number of minutes with recordings of echolocation and social calls of the soprano pipistrelle by month

Social calls were recorded from April to November (Fig. 2). The vast majority was recorded in August and September, with a few into October. In August and September, the numbers of social call minutes equalled nearly half the total number of pipistrelle echolocation recordings (Fig. 2). The denser area of increased activity in Fig. 1 from the middle of August to late September probably reflects the main mating season, with males patrolling back and forth both calling and hunting.

**Fig. 3:**
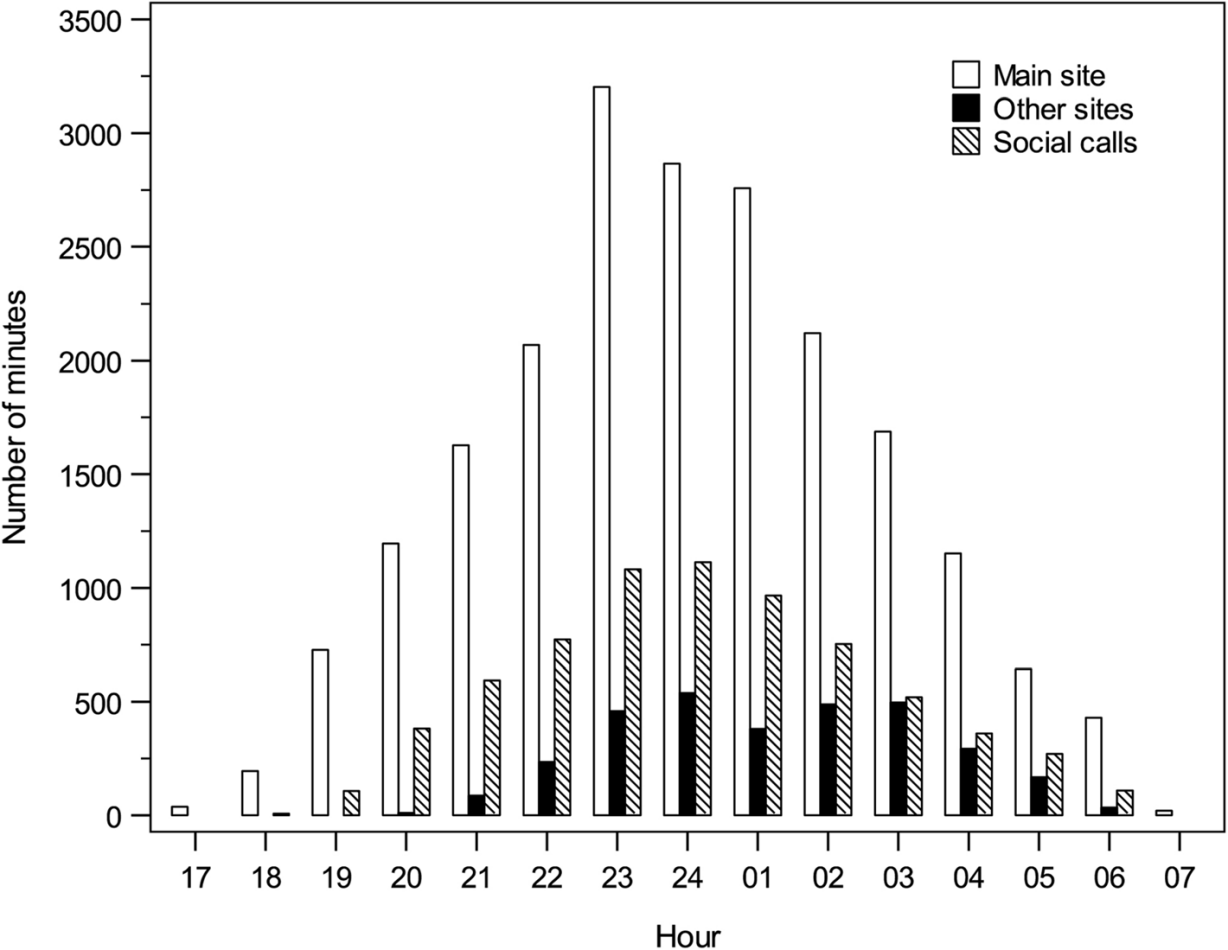
Number of minutes with recordings of the soprano pipistrelle by hour of the night. Presented are echolocation recordings at the main site, echolocation recordings at other sites and social calls at the main site

Most recordings were made around midnight, with social calls following the pattern of the echolocation recordings (Fig. 3). Because the pipistrelle’s first emergence tracked the time of sunset, they emerged progressively later until the summer solstice in June and thereafter progressively earlier until November (Fig. 1). In June, the total activity period of the pipistrelles was 5 h 47 min and in July 7 h 22 min (Table 1). This extended to up to about 12 h in September. At sites in Frafjord other than the main site, the recorded activity period was slightly longer in August (Table 1). The recordings from other sites were skewed slightly later in the night than those from the main site (Fig. 3). Both the absolute earliest record in the evening and the latest record in the morning were made in December 2019, at 1713 h on the 29th and at 0916 h on the 23rd, respectively. Both times were between sunset and sunrise.

## Discussion

This study confirmed that the soprano pipistrelle is an “early riser” compared to most other bats, utilizing the full night between sunset and sunrise [22]. Throughout the year, the bats closely tracked the timing of sunset and sunrise, most notably expanding their nightly activity period in late summer and autumn. They sometimes, but very rarely, started to fly before sunset, but hardly ever flew after sunrise. Consequently, the results from this study did not directly corroborate data from the northern limit of the soprano pipistrelle’s distribution in Norway at 62°N (25, 21] where they sometimes started to fly earlier in the evening relative to sunset. Petrzelková et al. [5] found that light levels at exit varied among roosts of the soprano pipistrelle, but were mostly below 600 lx. The common pipistrelle, on the other hand, appears to emerge from the roost considerably later. At 57° 13’N in Scotland, it emerged 35 min after sunset, in light levels of 15–35 lx [5, 8]. A similar result was found at 51° 45’N in England [11].

This study also confirmed that the soprano pipistrelle hibernated in Frafjord, even in sub-zero temperatures as does the common pipistrelle [31]. Hibernation in bat boxes has also been found further north in western Norway [32], and may be a regular strategy along the south-western coast of Norway where such boxes are available. However, their hibernation, or at least that of some individuals, was not as constant as expected. Flying bats could be encountered in any month of the year, although very rarely in the winter months November–March. Winter flights could involve both hunting and movement to a different hibernacula [26, 31]. In the mild winters of this region, the soprano pipistrelle appeared to have a flexible winter hibernation strategy. Since the harem in the bat box most likely consisted of one male and several females, it is unlikely that winter movements were restricted to males only. Among the seven bat species recorded in Frafjord, only the soprano pipistrelle flew in November–March (the other species found are *Pipistrellus nathusii*, *Eptesicus nilssonii*, *Myotis daubentonii*, *M. mystacinus*, *Vespertilio murinus* and *Nyctalus noctula*).

Although the exact dates are not known, it is likely that soprano pipistrelle nursery roosts in Frafjord existed from May to early July. The bats then moved to mating roosts (harems) which lasted until the end of September or early October. Winter hibernation lasted from late October to late April, and, at least in the case of the wood-concrete bat box, could occur as a continuation at the harem site. A few bats moved between different sites during the winter months and some also hunted on warmer nights.

The main season of soprano pipistrelle activity in Frafjord was from the last days of April to the middle of October. This is very similar to the common pipistrelle in England [11], but the two species had yet to be separated at the time of that study. It is also similar to *Pipistrellus* spp. in Northern Ireland [33]. At 50° 49′N in Germany, the common pipistrelle arrived at the roosts around the middle of May and roost switching by breeding females was common [14]. Maier [11] observed the first volant young around 8 July and, based on that date, estimated that parturition took place around 17 June. In Frafjord, many more recordings of the soprano pipistrelle were made in September than in any other month, which may largely have been due to male song flights. Consequently, the timing of the first flight of young bats could not be determined in this study. The increase in social calls found in August–September was similar but not identical, to the increase found in September–October in Northern Ireland [33, sensu 27]. In one study, the soprano pipistrelle foraged 204 min per night [34]. In Frafjord, the night was nearly 6 h long at its shortest in June, almost twice the length of that foraging time. Consequently, the soprano pipistrelle could follow a safe strategy of only flying when the sun was below the horizon, timing its activity in relation to sunset and sunrise. Although this meant some flying in twilight conditions, in the valley this was probably to some extent amended by a mountain shadow.

## Conclusions

Flying soprano pipistrelles were encountered in all months of the year, but their main active season was between late April and late October, or 6–7 months. Very few flights were recorded during December–March, although bats hibernating in a bat box came and left also at this time. Their nightly activity closely tracked sunset and sunrise, with very few recordings outside this period. This implied changes in the length of the bats’ hunting period throughout the season, being shortest at mid-summer when the energy demands on reproducing females should be large. Male song flights were recorded from April until November, with most recordings in August and September. At this time, males gather harems of females, and such groups may continue to use a bat box for example, well into the winter and hibernation period. It appears that this study is the first to study the activity of this species throughout the year and comprehensively relative to sunset and sunrise. This knowledge should be of importance to conservation and management, which need information both about seasonal activity, habitat preferences, breeding roosts and winter hibernaculas. In Norway, the soprano pipistrelle is classified as of least concern (LC), but little is known about population size or trend.

## Methods

**Fig. 4:**
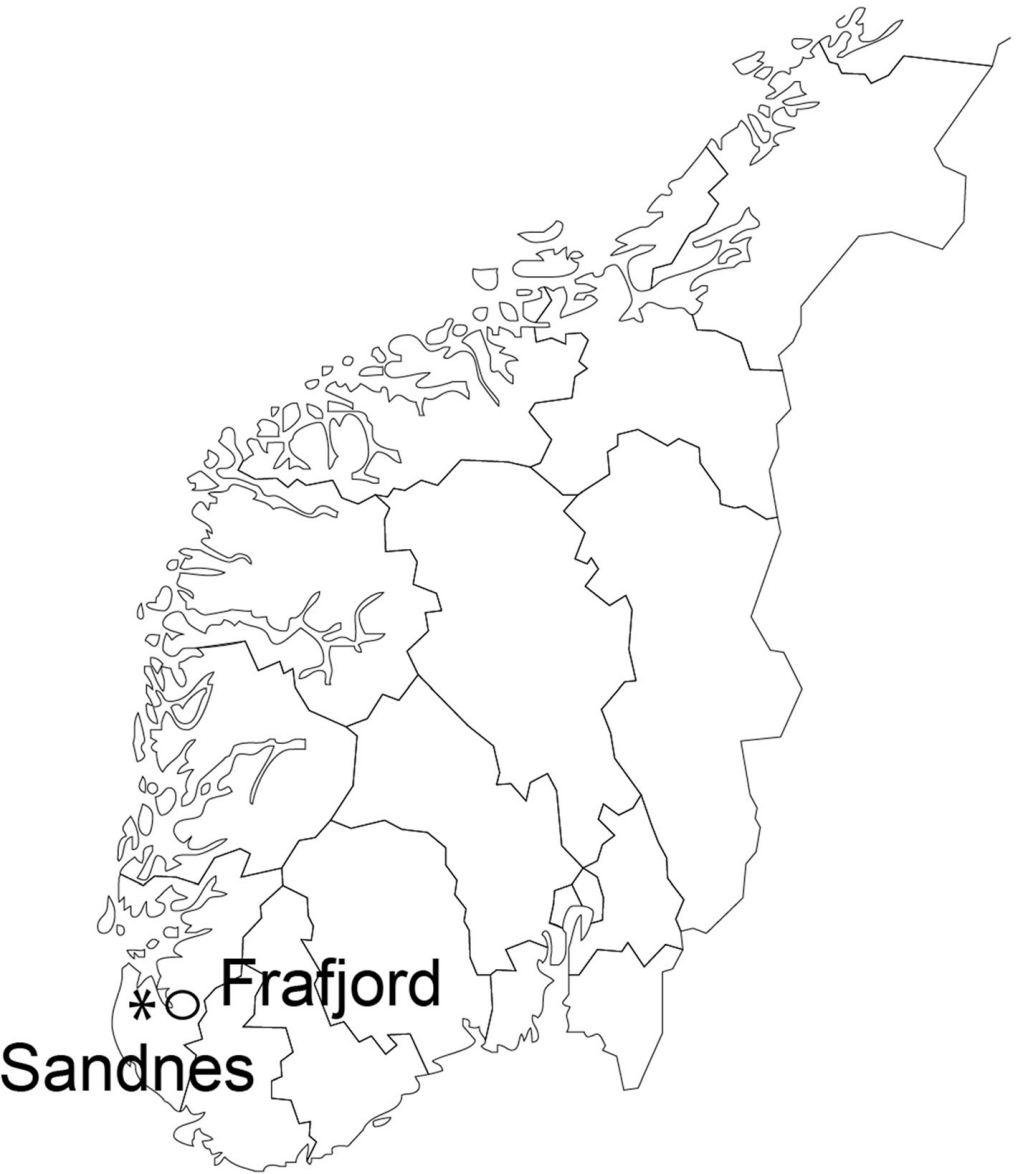
Map of South Norway with the locations Frafjord (○) and Sandnes (*) marked

Soprano pipistrelles were studied in Frafjord (58° 50′N 6° 18′E). This is a small valley with steep sides running west to east in Rogaland county, on the SW coast of Norway (Fig. 4). It has a coastal climate with a large annual rainfall, mild winters and little snow.

Bat sounds were recorded with an ultrasound recorder, Wildlife Acoustics SM2Bat (Wildlife Acoustics, USA). This recorder was in operation over more than a year, from 26 April 2018 to 27 June 2019, at a single site termed the main site (a farm in the central part of the valley, Additional file 1), although the recordings were not fully contiguous. Data are missing from April and May 2019 due to a faulty memory card, hence, this period was covered in 2020 (21 March to 16 June).. The microphone was placed about 3 m above ground, on the farmhouse and facing a garden, a small road and a field.

In June–July 2016, August 2017, late June–July 2019 and July 2020, ultrasound recordings were made at various other sites (termed “other sites”) surrounding and including the main site described above (Additional file 1). This was an attempt to map specific areas particularly used by the bats, as well as their activity. The distance between the outermost sites was 2.7 km. Additionally, some recordings combined with observations were made in December 2019 to January 2020, these are only included in Table 2.

Generally, the recorder was programmed to start 0.5 or 1.0 h before sunset and ran until the same interval after sunrise (bats were never seen flying in daylight hours). From October to December 2018, the nightly recording program was reduced to around 9 h from sunset, i.e. the recorder stopped before sunrise, to limit the huge amount of work needed to go through all the files generated. In 2020, the recordings started 1 h before sunset and ran until 1 h after sunrise.

Because seven bat species have been recorded in Frafjord, all recordings had to be first identified to species. Files were first automatically scanned in Song Scope (Wildlife Acoustics, USA) using a species-specific identifier. The identified bat emissions were then manually checked and the files further searched by looking at spectrograms throughout the file and species identified. Every minute during which the sound of a soprano pipistrelle was identified represented one case, so that the sample size is the number of minutes in which a bat was identified (Additional file 2). Social calls (characteristic low-frequency calls, e.g. 29) were identified to the minute by the same procedure. Multiple soprano pipistrelles recorded simultaneously were ignored and recorded as 1 min.

Principally, pipistrelle echolocation calls above 48 kHz were identified to be from a soprano pipistrelle [28, 35]. A few recordings of Nathusius’ pipistrelle *Pipistrellus nathusii* were also made in Frafjord, but this species appeared to be a rare visitor. Soprano and Nathusius’ pipistrelles can relatively safely be distinguished by the frequency of their echolocation calls [28, 36]. The common pipistrelle was most likely absent in Frafjord.

Light level data were not collected in Frafjord, but some data collected at a site 32 km from Frafjord, at the city of Sandnes (58° 51′N 5° 44′E, Fig. 4), in 2007 were used for comparison. This location is open space, but surrounded by houses. These data were collected using a Pace Scientific Inc. Pocket logger XR440, with the sensor placed about 5 m above the ground. Recordings were made every 10 min through all 24 h. This only gives an approximation of the levels in Frafjord, and is only used to indicate maximum possible levels at sunset and sunrise.

The timing of the sunset, sunrise and sun at its lowest inclination angle in the north were downloaded from https://www.timeanddate.no/ (September 2019) for Sandnes (Fig. 4). All data were adjusted to Norwegian summer time (daylight saving time, UTC + 2 h). Because bat activity was centred around midnight, the presentations are based on “night” rather than “day”. Each night crosses two dates, but was assigned to its start date. Because sunset and sunrise are only 2 min earlier in Frafjord than in Sandnes, this small difference was ignored.

The pipistrelles hunted so regularly at the farm that a roost most likely existed nearby. Two roosts were known some 300 m away, but neither was a nursery roost with young. One was in a wood-concrete bat box mounted in a tree, with an open bottom that made inspection possible without disturbing the bats. Sometimes the bat box and the other roost was inspected from below with a small torch and the number of pipistrelles counted. These counts were only approximate, as it was difficult to separate individual bats when they clumped together. The combined number of pipistrelles in these roosts was small, maximum around ten bats. Close to the two roosts mentioned, a third roost site was found in a stack of wooden and polystyrene boxes, about 1 m high. In the autumn 2019, one dead pipistrelle was found along with a large number of droppings indicating that this unlikely site may have been a nursery roost in the summer 2019.

## Supplementary Information

**Additional file 1.** Topographical map of the Frafjord valley, southwest Norway, with all locations were the Wildlife Acoustics SM2Bat recorder was applied. Circle: main site, *: other sites.

**Additional file 2 **Dataset by month and year for *Pipistrellus pygmaeus* recorded in Frafjord, Norway. (XLS 39 kb)

## Data Availability

The dataset supporting the conclusions of this article is included within the article and in one additional file.
