# Supplementary figures and images for: Activity patterns of the soprano pipistrelle Pipistrellus pygmaeus throughout the year in southern Norway
Source: BMC Zool. 2021 Feb 9;6:1. doi: 10.1186/s40850-021-00065-x (PMC10127296; doi:10.1186/s40850-021-00065-x)

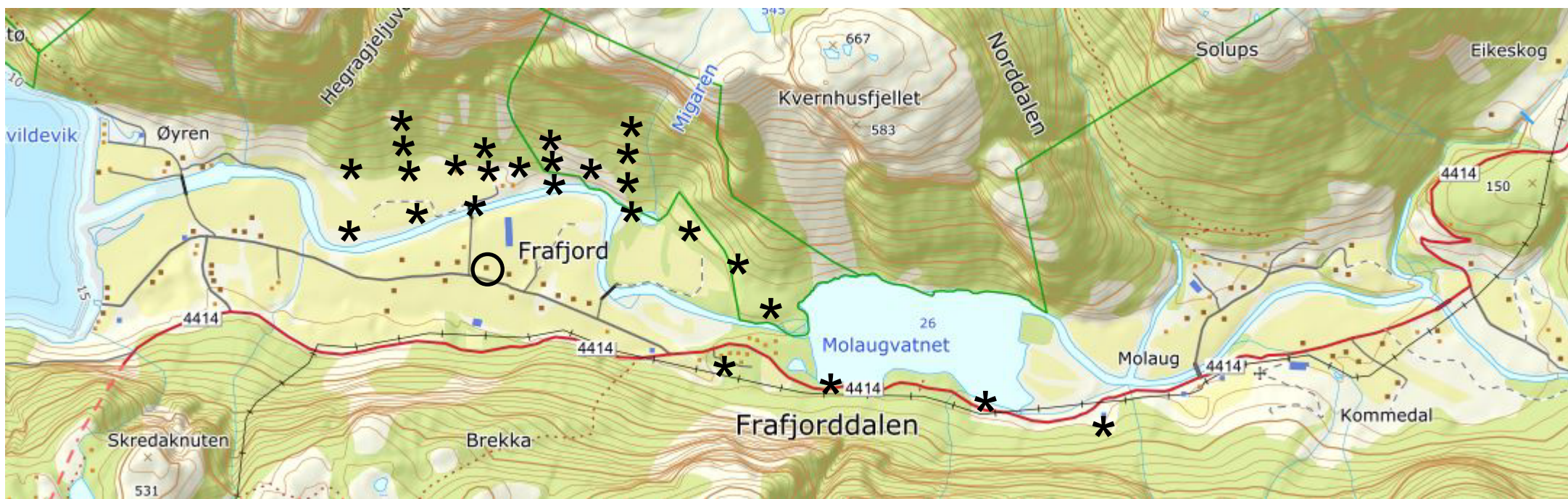

200 m

Supplement: Supplementary file 1 — Additional file 1. Topographical map of the Frafjord valley, southwest Norway, with all locations were the Wildlife Acoustics SM2Bat recorder was applied. Circle: main site, *: other sites. [file 40850_2021_65_MOESM1_ESM.pdf]
